# Supplementary material for: A Novel Role of BIRC3 in Stemness Reprogramming of Glioblastoma
Source: Int J Mol Sci. 2021 Dec 28;23(1):297. doi: 10.3390/ijms23010297 (PMC8745052; doi:10.3390/ijms23010297)
Supplement: Supplementary file 1 [file ijms-23-00297-s001.zip › ijms-1482956-supplementary.pdf]

Figure S1

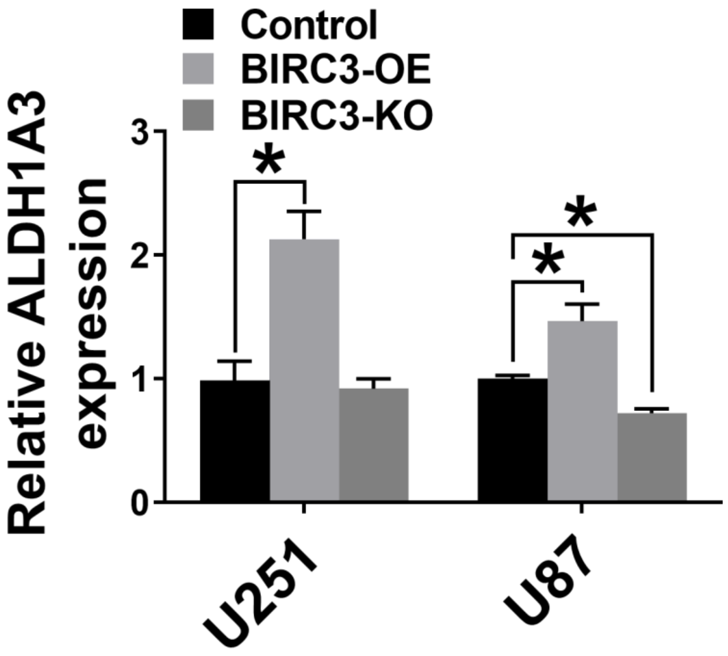

Fig S1. ALDH1A3 mRNA expression analyzed by real-time PCR in U251/U87 control, BIRC3-OE and BIRC3-KO cells. n=3, * $p<0.05$.

Figure S2

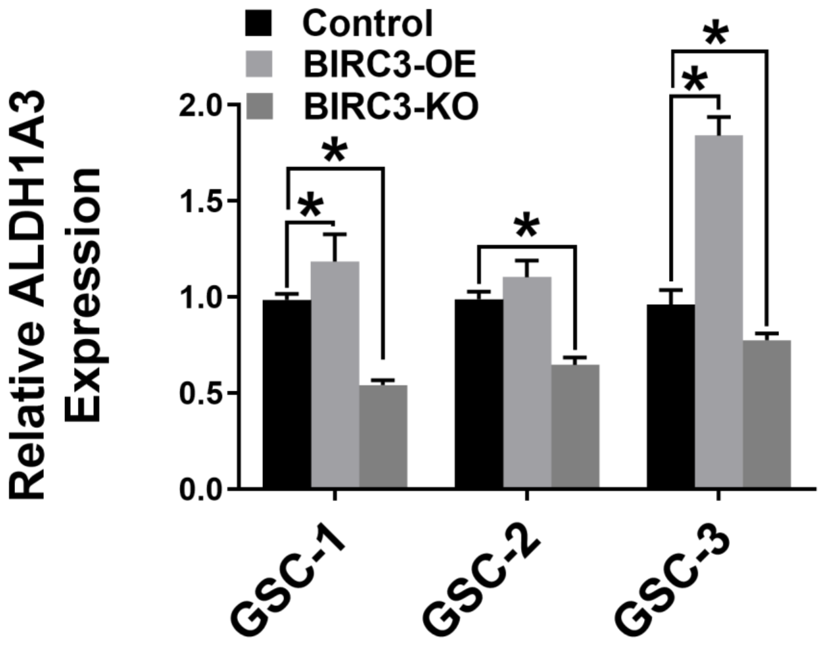

Fig S2. ALDH1A3 mRNA expression analyzed by real-time PCR in control, BIRC3-OE and BIRC3-KO GSCs. n=3, * p<0.05.

Figure S3

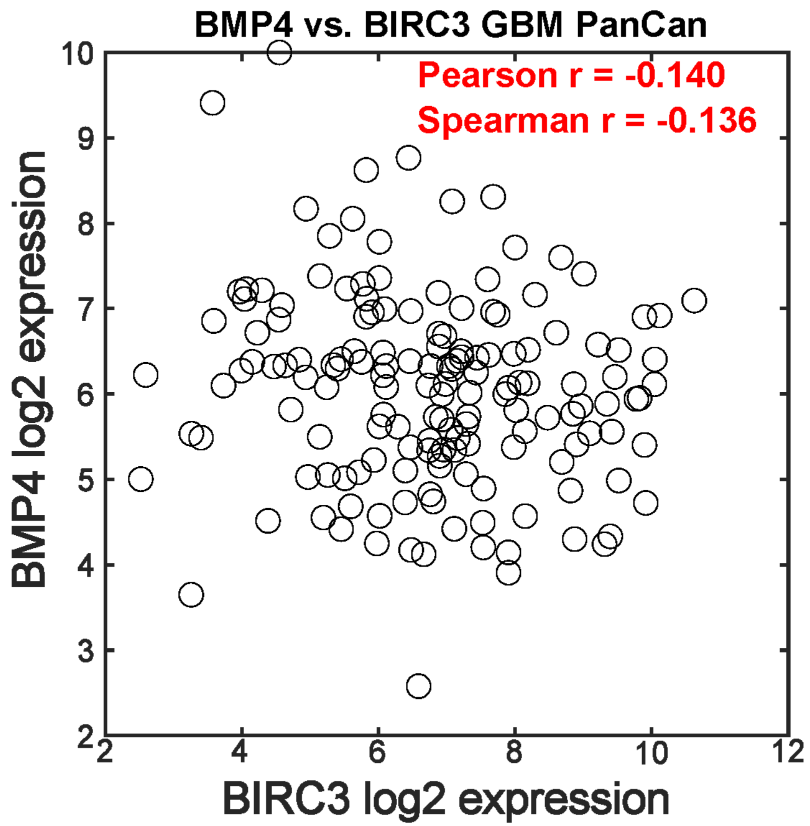

Fig S3. Scatterplots of correlation between BIRC3 and BMP4 expression observed in TCGA GBM PanCan datasets.

Raw Fig1-2

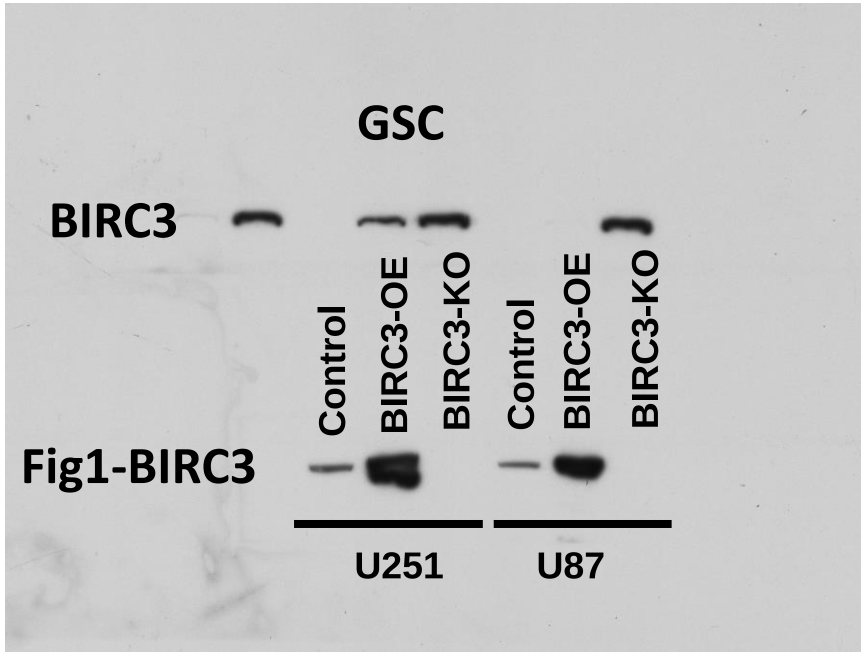

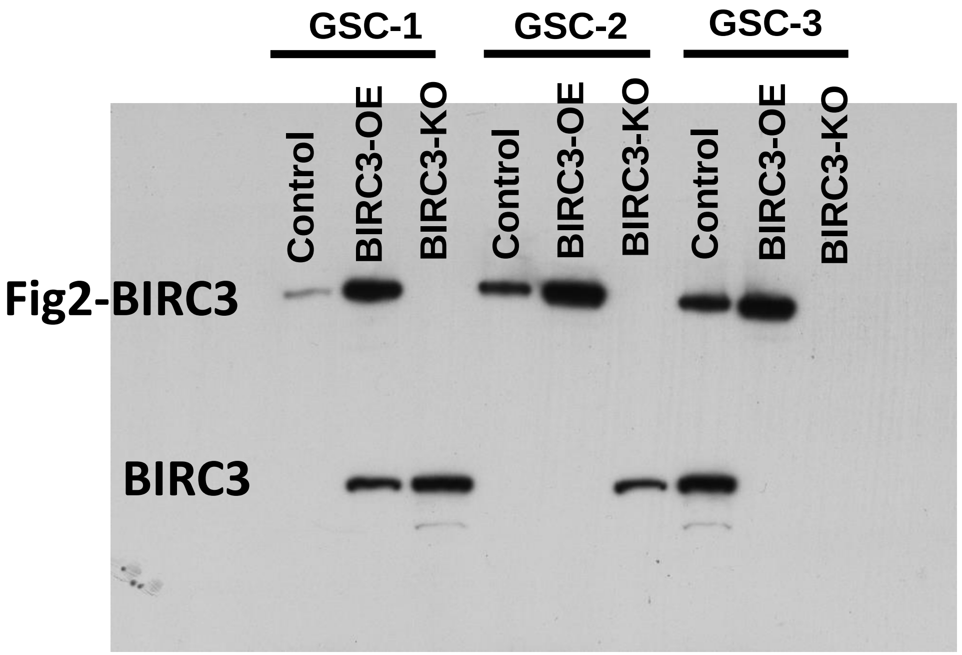

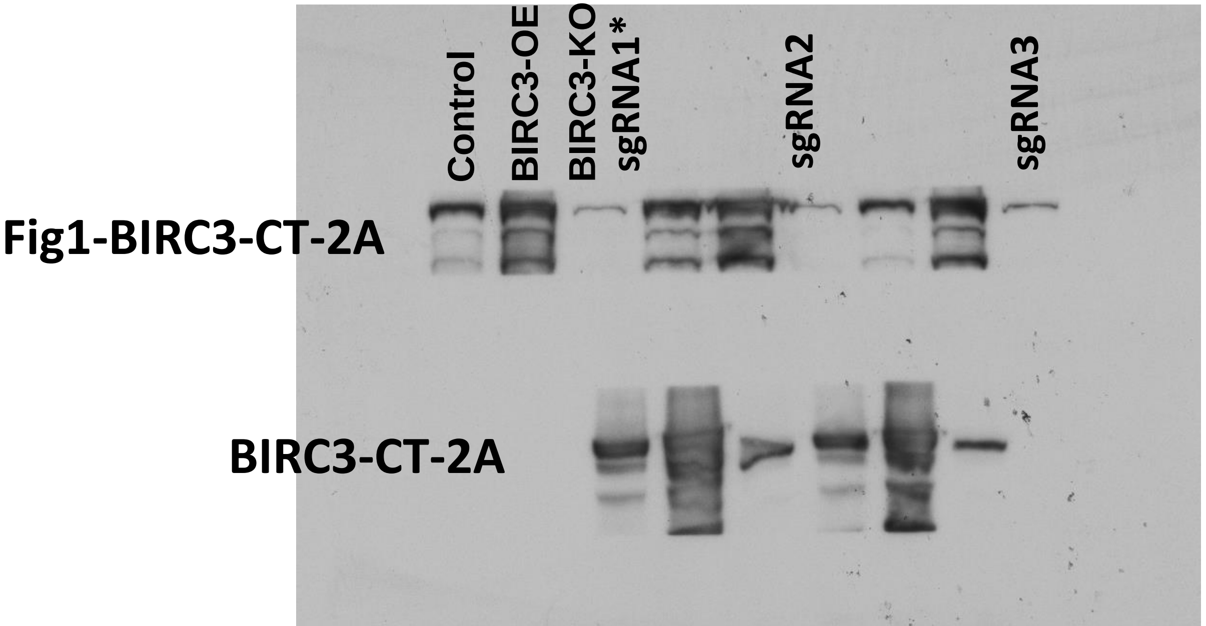

* sgRNA1 is the sgRNA used in the manuscript for CT-2A cell line

Raw Fig1-2

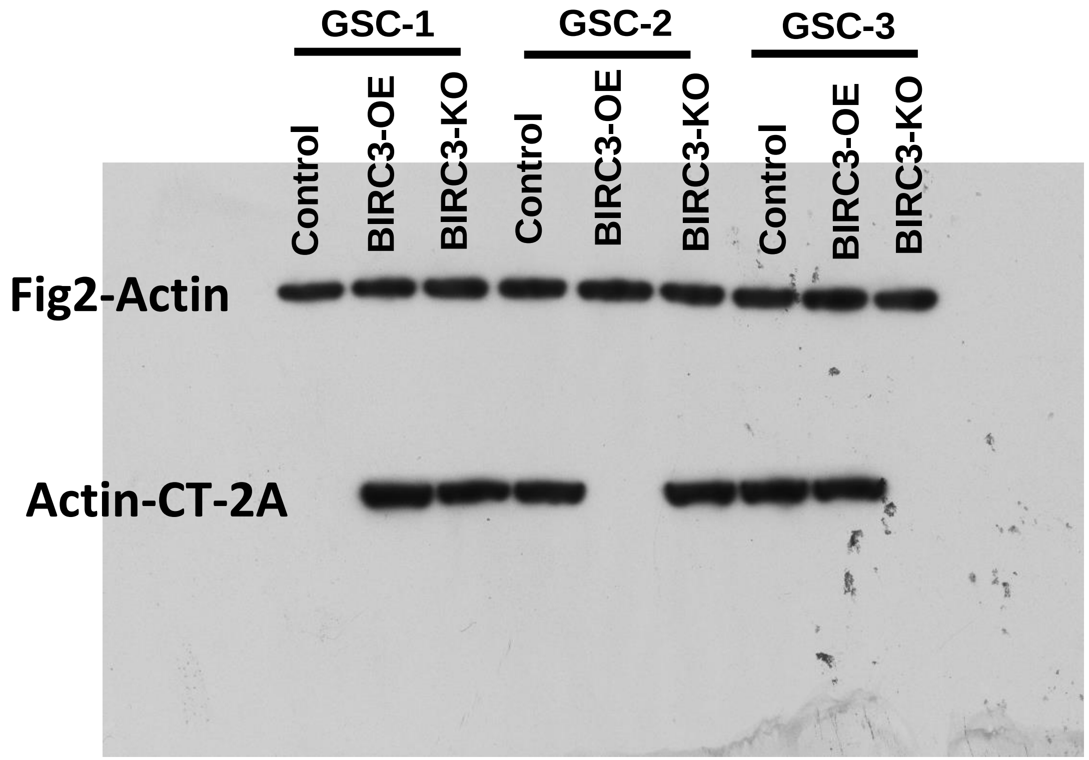
GSC-1
GSC-2
GSC-3
Control
BIRC3-OE
BIRC3-KO
Control
BIRC3-OE
BIRC3-KO
Control
BIRC3-OE
BIRC3-KO
Fig2-Actin
Actin-CT-2A

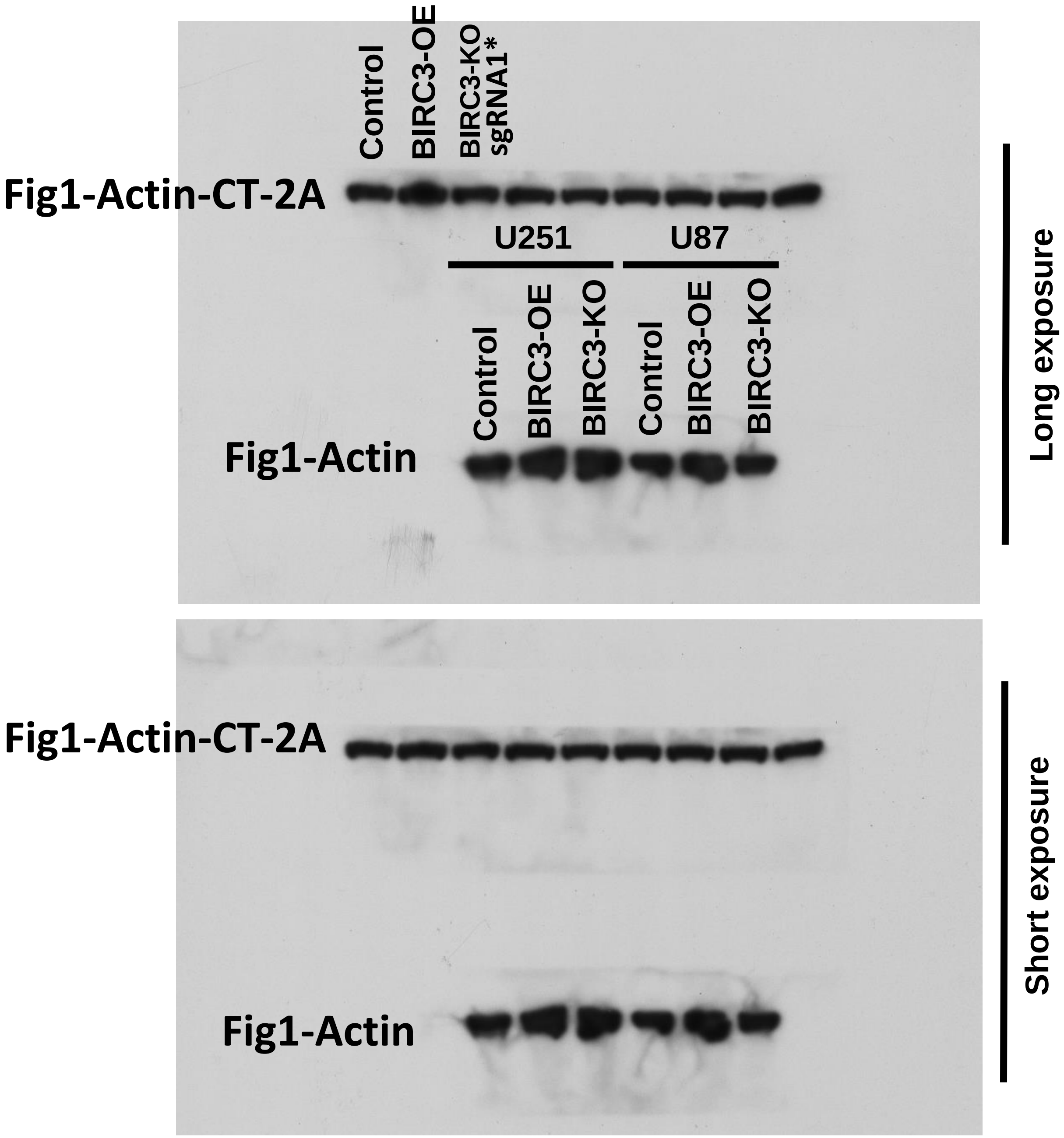
Control
BIRC3-OE
BIRC3-KO sgRNA1*
Fig1-Actin-CT-2A
U251
U87
Control
BIRC3-OE
BIRC3-KO
Control
BIRC3-OE
BIRC3-KO
Fig1-Actin
Long exposure
Fig1-Actin-CT-2A
Fig1-Actin
Short exposure

Raw Fig3-4

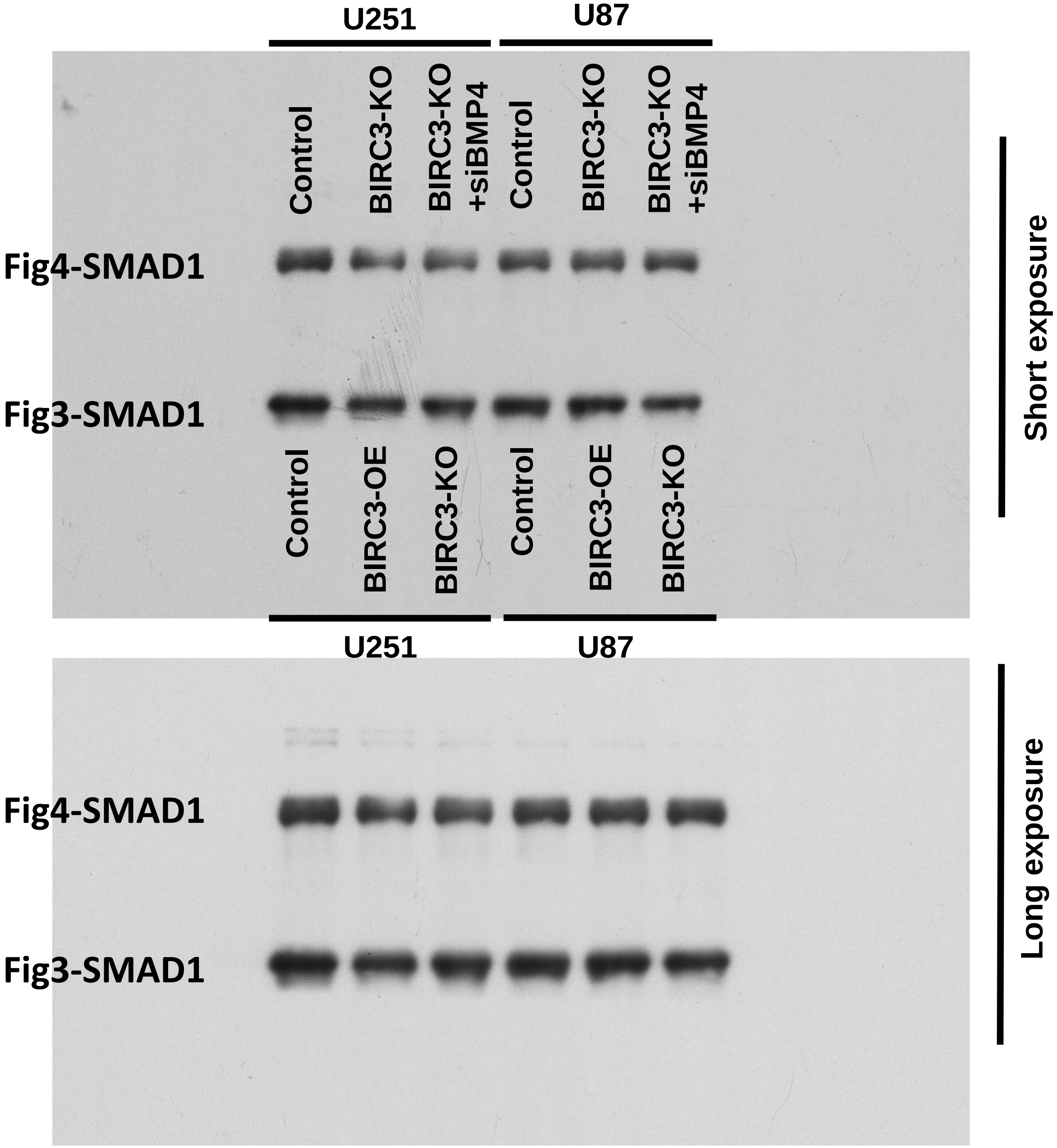
U251
U87
Control
BIRC3-KO
BIRC3-KO +siBMP4
Control
BIRC3-KO
BIRC3-KO +siBMP4
Fig4-SMAD1
Fig3-SMAD1
Control
BIRC3-OE
BIRC3-KO
Control
BIRC3-OE
BIRC3-KO
U251
U87
Short exposure
Fig4-SMAD1
Fig3-SMAD1
Long exposure

Raw Fig3-4

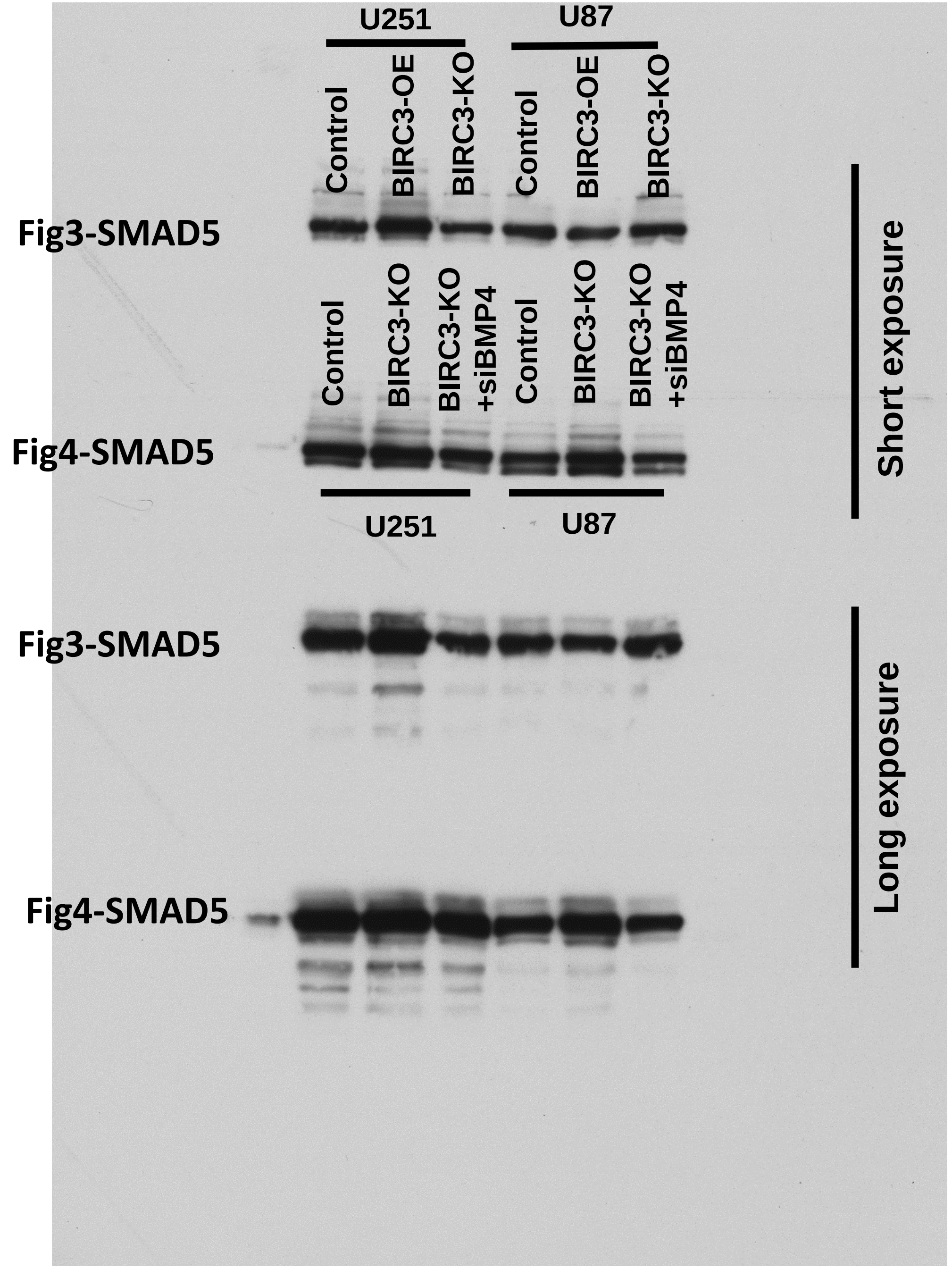
U251
U87
Control
BIRC3-OE
BIRC3-KO
Control
BIRC3-OE
BIRC3-KO
Fig3-SMAD5
Control
BIRC3-KO
BIRC3-KO +siBMP4
Control
BIRC3-KO
BIRC3-KO +siBMP4
Fig4-SMAD5
U251
U87
Short exposure
Fig3-SMAD5
Fig4-SMAD5
Long exposure

Raw Fig3-4

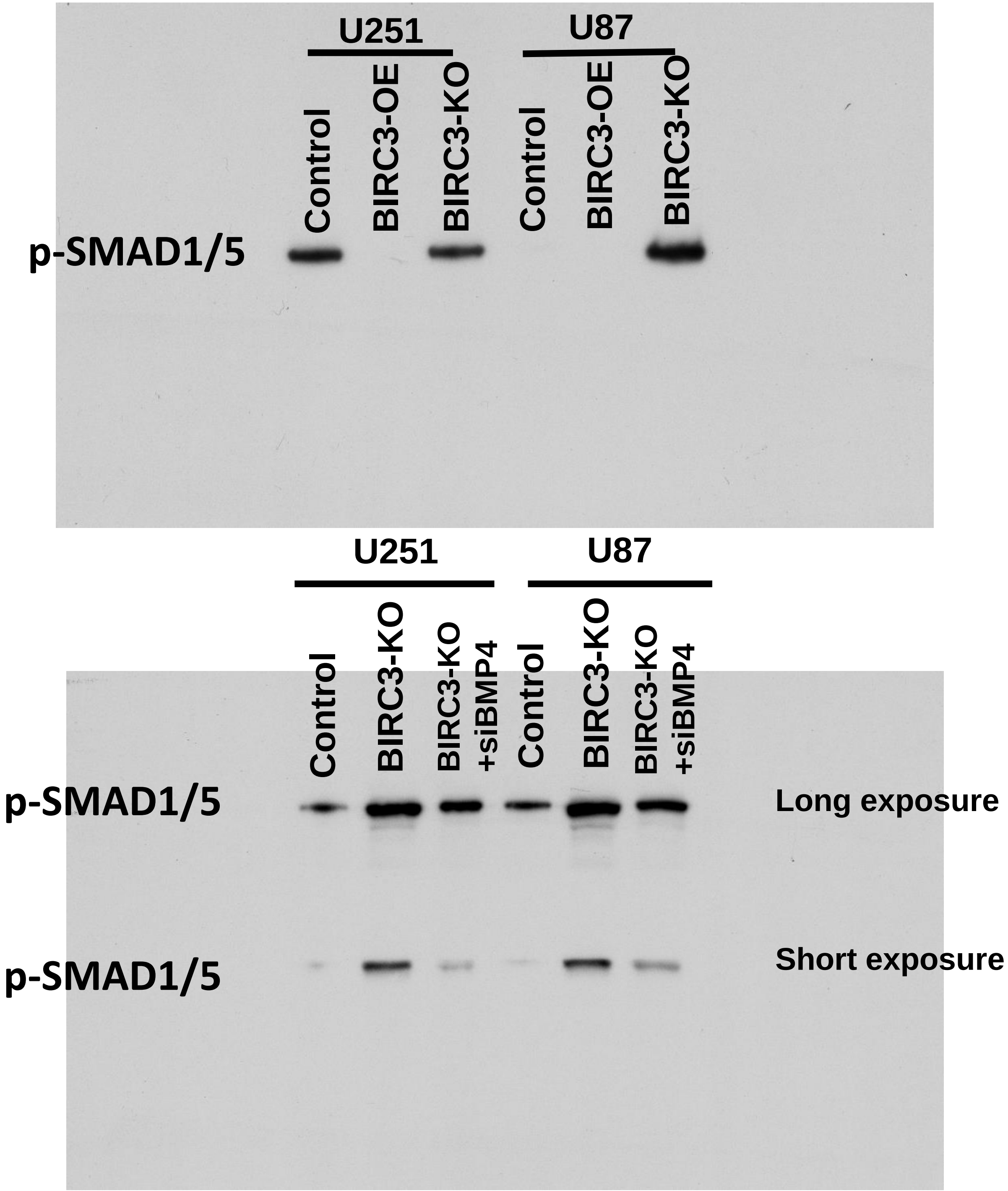
U251
U87
Control
BIRC3-OE
BIRC3-KO
Control
BIRC3-OE
BIRC3-KO
p-SMAD1/5
U251
U87
Control
BIRC3-KO
BIRC3-KO +siBMP4
Control
BIRC3-KO
BIRC3-KO +siBMP4
p-SMAD1/5
Long exposure
p-SMAD1/5
Short exposure

Raw Fig3-4

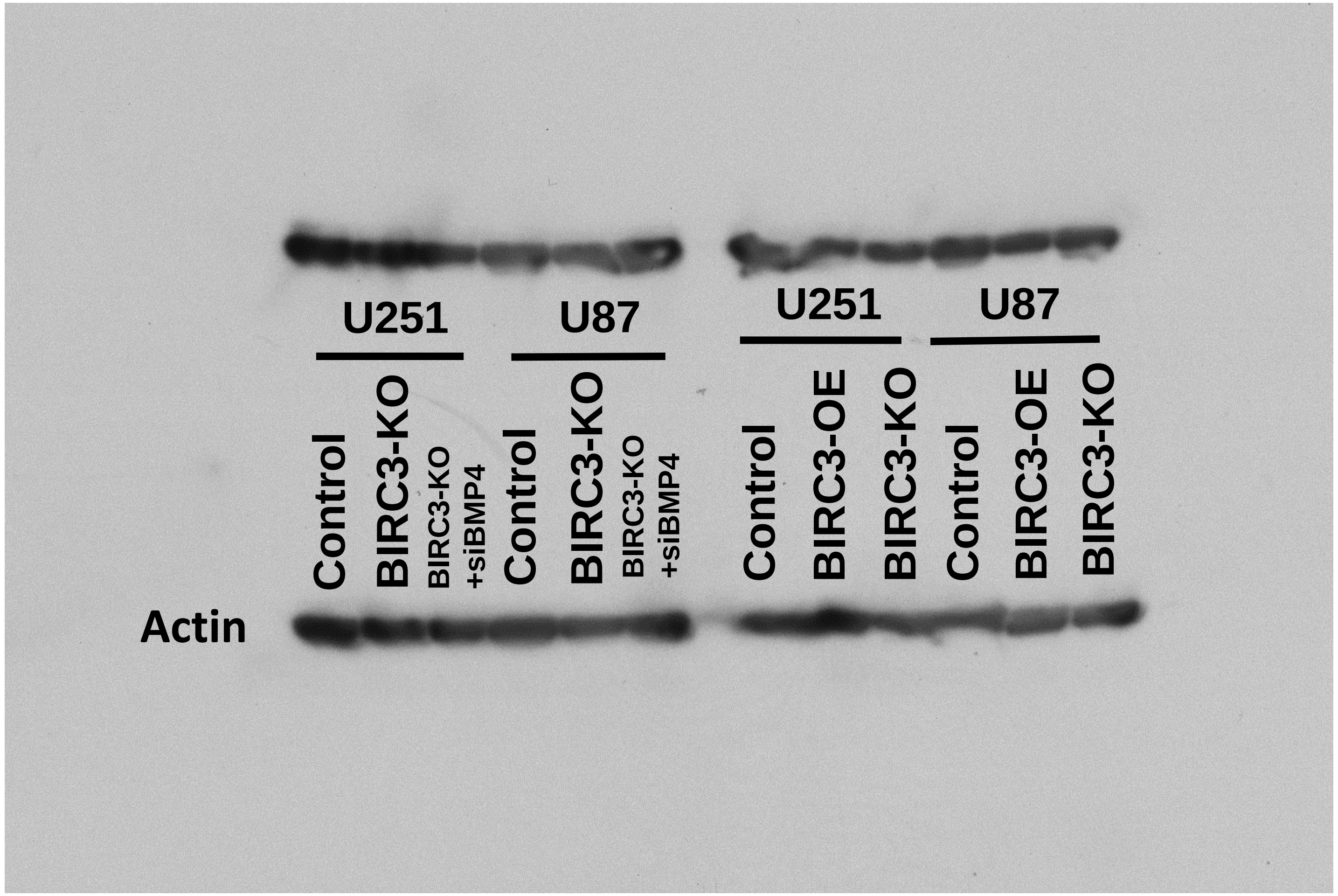
U251
U87
U251
U87
Control
BIRC3-KO
BIRC3-KO +siBMP4
Control
BIRC3-KO
BIRC3-KO +siBMP4
Control
BIRC3-OE
BIRC3-KO
Control
BIRC3-OE
BIRC3-KO
Actin
